# Radiographer Preliminary Image Evaluation Accuracy in Interpreting Paediatric Trauma Radiographs

**DOI:** 10.1002/jmrs.876

**Published:** 2025-03-05

**Authors:** Teresa Poon, Michael Neep, Therese Gunn

**Affiliations:** ^1^ Department of Medical Imaging Logan Hospital Meadowbrook Queensland Australia; ^2^ School of Clinical Sciences, Queensland University of Technology, Gardens Point Brisbane Australia

**Keywords:** child, computer‐assisted image interpretation, medical audit/statistics and numerical data, radiography/statistics and numerical data, sensitivity and specificity

## Abstract

**Introduction:**

Radiographer preliminary image evaluation (PIE) is a patient safety mechanism where radiographers provide a written comment describing potential pathology in radiographs they have acquired. This can assist emergency referrers in making a diagnosis when a radiologist's report is unavailable. The aim of this study was to evaluate the accuracy of radiographer PIE in interpreting paediatric trauma radiographs in an Australian emergency department.

**Methods:**

A randomised sample of paediatric radiographic examinations (aged 16 years and under) from January 2022 to June 2023 was retrospectively reviewed. The anatomical regions reviewed included the appendicular and axial skeleton, chest and abdomen. The PIE for each examination was compared to the radiologist report to indicate if the radiographer's evaluation was a true negative/positive or false negative/positive value. This was used to calculate mean sensitivity, specificity and diagnostic accuracy. Discrepant PIE interpretations were further investigated.

**Results:**

A total of 498 PIEs were reviewed. The overall accuracy, sensitivity and specificity were 93.3%, 84.3% and 98.1%, respectively. Cases with no participation and those marked as unsure for pathology represented 0.4% and 2.6% of the sample, respectively. The extremities were identified as a region frequently misinterpreted.

**Conclusion:**

Radiographers in this study maintained a high diagnostic accuracy in interpreting paediatric radiographs. PIE may complement the emergency referrer's diagnosis when a radiologist report is unavailable to promote appropriate and timely treatment for paediatric patients in the emergency department. Further research with a larger sample may support targeted training to improve PIE performance in regions frequently misinterpreted.

## Graphical abstract

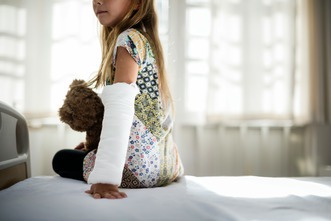

## 1 Introduction

Plain radiography is frequently used in emergency departments (EDs) to help inform the diagnosis and management of paediatric patients presenting with trauma [1]. However, the radiologist report for radiographic examinations is often unavailable at the time of patient treatment in adult and paediatric EDs [2, 3, 4]. The radiologist report provides a formal communication of imaging findings to aid emergency clinicians in clinical decision making, and it represents the international ‘gold standard’ in interpreting radiographs [1, 5]. In order to effectively influence the clinical management of the patient, radiologist reports for patients in the ED should be available in a timely manner to facilitate patient admission, discharge or transfer within 4 h [6]. In the absence of a radiologist report, emergency clinicians are required to make treatment decisions based on their own interpretation of the radiographs, which may contribute to diagnostic errors in the ED [2, 4]. Emergency clinicians who interpret radiographs may be medical officers ranging in experience from interns to consultants, nurse practitioners and emergency physiotherapist practitioners [2, 7]. Inexperienced emergency clinicians may have limited or variable experience in interpreting radiographs, particularly paediatric radiographs [2, 4, 8, 9]. The interpretation of paediatric radiographs presents additional challenges due to differences in the appearances of developing anatomy, pathology and paediatric injury patterns, which vary by age [9, 10]. Accurate interpretation requires awareness of diseases distinct to the paediatric population and of normal developmental anatomy, including growth plates and ossification centres [9, 10]. The literature has highlighted that paediatric radiographs are interpreted less accurately by emergency clinicians than adult radiographs, with a reported diagnostic error rate of 5%–15% in interpreting paediatric musculoskeletal radiographs [2, 8, 9, 11]. Errors in interpreting radiographs can lead to delayed optimal treatment, unnecessary morbidity, increased patient uncertainty, increased costs to the healthcare system and malpractice allegations [8, 9, 12]; thus, innovations are necessary to maintain patient safety in the ED [13].

Radiographer preliminary image evaluation (PIE), also called preliminary clinical evaluation in the United Kingdom (formally referred to as radiographer commenting), is a patient safety mechanism that aims to improve patient care by reducing errors in radiographic interpretation [2, 14]. A PIE is a brief written comment provided by the radiographer immediately after completing the radiographic examination, which describes the presence or absence of potential pathology [2, 14]. In most institutions, a PIE is documented prior to the generation of the radiologist report, as the PIE is completed at the time of imaging. A PIE does not replace the radiologist report; rather, it provides a timely and documented communication to emergency clinicians, highlighting potential abnormalities to support patient treatment decisions in the absence of a definitive radiologist report [14].

Several studies have investigated the performance of radiographer PIE in clinical practice, reporting an overall accuracy ranging from 88% to 92% [2, 13, 15, 16, 17, 18]. A salient study published in 2023 found that when combined with the radiographer's interpretation, the emergency clinician's interpretation of radiographic examinations can be more accurate than the emergency clinician's interpretation in isolation [2]. However, the majority of studies investigated the performance of PIE in interpreting adult examinations only [16, 17, 18] or did not differentiate between paediatric and adult examinations during data analysis [13, 15]. There is currently a paucity of studies that explore radiographer PIE in the paediatric population [2]; thus, the present study aimed to address this knowledge gap. The purpose of the present study was to investigate the accuracy of radiographer PIE in interpreting paediatric trauma radiographs in clinical practice.

## 2 Method

### 2.1 Ethics

Ethical approval was granted by the Metro South Hospital and Health Service Human Research Ethics Committee and the Queensland University of Technology Research Governance and Integrity Team. No personal identifying information was collected during this study.

### 2.2 Study Design

This retrospective clinical audit was conducted over an 18‐month period from January 2022 to June 2023.

### 2.3 Study Setting and Participants

This study was undertaken in a metropolitan hospital in Southeast Queensland that provides services for both paediatric and adult patients. The PIE service at this hospital was implemented 7 years prior to the commencement of the present study and was operational 24 h a day and 7 days a week within the ED throughout the study period. All radiographers were required to participate in the PIE service when working in the ED, and radiographers ranged from being newly qualified to having over 15 years of experience. All radiographers completed in‐house X‐ray interpretation training modules within 10 weeks of commencing participation in the PIE service. At this institution, the scope of radiographic examinations in ED that required a radiographer PIE was referrals that queried acute bony fractures, dislocations or subluxations, foreign bodies, elbow joint effusions, lipohaemarthrosis of the knee, pneumothorax and pneumoperitoneum. Examinations not querying any of these pathologies were considered outside of scope and were not included in the audit.

### 2.4 Sample Size Calculation

The key objectives were to obtain a random sample that represented a variety of radiographic examinations performed at varying times of day, over a range of days during the week and by a variety of radiographers. As part of the routine monthly PIE audit at the study site, 100 examinations were audited each month. Randomisation was applied by progressively selecting from a list of alphabetised surnames; for example, if 100 examinations were audited with the surnames beginning with A to C in 1 month, then in the following month, the audited examinations would start from patients with the surname beginning with D, and so on. The audited examinations for each month during the present study period were filtered for paediatric patients (≤ 16 years), resulting in 498 paediatric examinations for the present study. In the span of 1 year, from July 2022 to June 2023, 9151 paediatric x‐ray examinations were performed in the ED. Given a recommended sample size of 385 (confidence level 95%, ± 5% confidence interval) calculated using a published audit size calculator, the sample size of this study was considered suitable [19].

### 2.5 Procedure

Each PIE comment was compared with the radiologist report and assigned an evaluation category, in line with the following categories used in previous literature: [2, 13, 15]

- True positive (TP): All acute abnormalities reported by the radiologist were correctly identified and described in the PIE.
- True negative (TN): The PIE agreed with the radiologist report on the absence of acute abnormality.
- False positive (FP): The PIE interpreted and described an appearance as abnormal; however, no acute abnormality was reported by the radiologist.
- False negative (FN): The PIE interpreted the examination as normal; however, the radiologist's report stated that an acute abnormality was present.
- TP/FN: Assigned when multiple acute abnormalities were described in the radiologist report, and the PIE correctly described a pathology but failed to describe all abnormalities, or the radiographer identified the correct abnormality but provided insufficient description (type of injury, site and side). For the calculation of sensitivity, specificity and accuracy, a TP/FN score was assigned 0.5 for the TP category and 0.5 for the FN category.
- Unsure: The PIE indicated that the radiographer was unsure.
- No participation: No PIE comment was provided on an examination within scope.

### 2.6 Data Analysis

The 498 cases were entered by the auditing radiographer into a Microsoft Excel spreadsheet recording the anatomical region imaged, the patient's age and the category of the PIE. Conventional descriptive statistics were used to describe patient age demographics. The data were grouped by age and anatomical region for analysis. The categories of PIE were analysed to determine the distribution of PIE results within groups and to calculate overall sensitivity, specificity and accuracy. Further analysis was undertaken to identify the anatomical regions with the highest number of FNs, FPs and TP/FNs.

Prior to analyses, inter‐rater and intra‐rater reliability were calculated on a subset of the sample to demonstrate the reliability of the audit methodology. This involved an additional auditor and the original auditor remarking 10% of the total sample (*n* = 50). Favourable inter‐rater reliability (kappa < 0.81 for all cases) and intra‐rater reliability (kappa < 0.89 for all cases) were calculated, consequently indicating a reliable audit process.

## 3 Results

**FIGURE 1:**
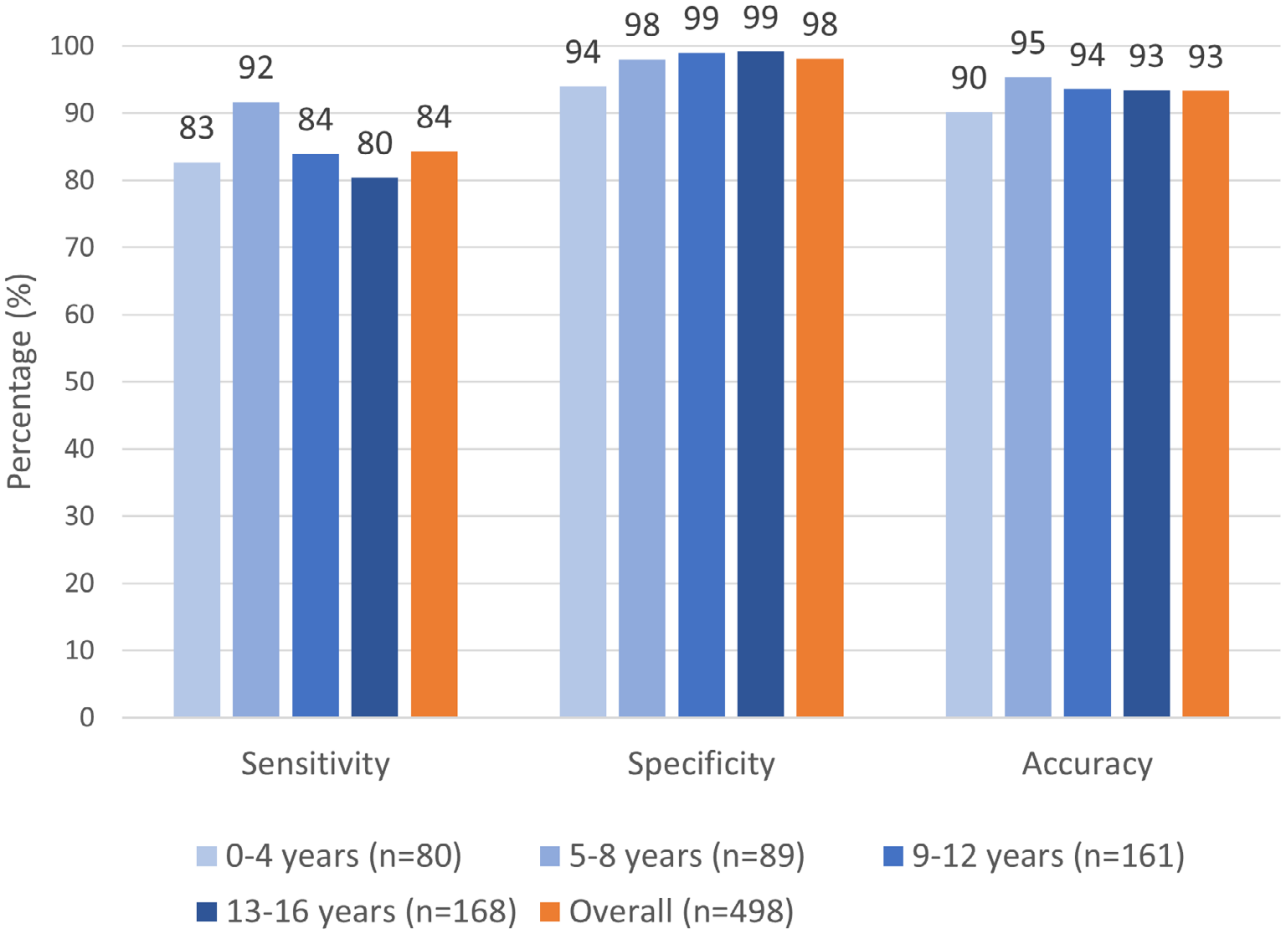
Results of sensitivity, specificity and accuracy analysis within age groups.

A total of 498 PIEs were reviewed. Table 1 presents a breakdown of the range of audited examinations. The overall distribution of PIE categories is presented in Table 2. The PIE service demonstrated a sensitivity, specificity and accuracy of 84.3%, 98.1% and 93.3%, respectively. Sensitivity, specificity and accuracy between age groups are presented in Figure 1. The overall PIE accuracy, inclusive of 13 (2.6%) unsure and 2 (0.4%) non‐participation cases, was 90.5%. The anatomical regions with the most unsure interpretations were the hand and fingers (*n* = 4), wrist (*n* = 2), elbow (*n* = 2) and forearm (*n* = 2). Non‐participation PIEs were recorded for two examinations, which both queried foreign bodies.

**TABLE 1:** Breakdown by anatomical region and age group.

Anatomical region	Accuracy of comment								
	Age (years)	TP^a^	TN^b^	FP^c^	FN^d^	TP^a^/FN^d^	Unsure	No participation	Total
Ankle	0–4	0	2	0	0	0	0	0	2
	5–8	0	6	0	0	0	1	0	7
	9–12	3	14	0	2	0	0	0	19
	13–16	5	21	0	1	1	0	0	28
	Total	8	43	0	3	1	1	0	56
Chest/Abdomen	0–4	2	10	0	0	0	0	1	13
	5–8	2	5	0	0	0	0	0	7
	9–12	0	6	0	0	0	0	0	6
	13–16	1	8	0	0	0	0	0	9
	Total	5	29	0	0	0	0	1	35
Elbow	0–4	6	1	1	1	0	1	0	10
	5–8	5	1	0	1	1	1	0	9
	9–12	1	3	0	1	0	0	0	5
	13–16	4	3	0	0	1	0	0	8
	Total	16	8	1	3	2	2	0	32
Femur	0–4	0	0	0	0	0	0	0	0
	5–8	0	0	0	0	0	0	0	0
	9–12	0	0	0	0	0	0	0	0
	13–16	0	1	0	0	0	0	0	1
	Total	0	1	0	0	0	0	0	1
Foot/Toes	0–4	0	7	0	0	0	0	0	7
	5–8	1	11	0	0	0	0	0	12
	9–12	8	15	0	0	0	1	0	24
	13–16	5	8	0	1	0	0	0	14
	Total	14	41	0	1	0	1	0	57
Forearm	0–4	5	8	1	1	1	1	0	17
	5–8	15	9	0	0	1	0	0	25
	9–12	12	11	0	0	0	1	0	24
	13–16	2	5	0	0	1	0	0	8
	Total	34	33	1	1	3	2	0	74
Hand/Fingers	0–4	3	6	1	1	0	0	0	11
	5–8	3	7	0	1	0	1	0	12
	9–12	4	14	0	2	0	3	0	23
	13–16	13	27	1	4	0	0	1	46
	Total	23	54	2	8	0	4	1	92
Humerus	0–4	0	2	0	0	0	0	0	2
	5–8	0	0	0	0	0	0	0	0
	9–12	0	3	0	0	0	0	0	3
	13–16	0	0	0	0	0	0	0	0
	Total	0	5	0	0	0	0	0	5
Knee	0–4	0	1	0	0	0	0	0	1
	5–8	0	4	0	0	0	0	0	4
	9–12	1	6	0	0	0	0	0	7
	13–16	0	15	0	1	0	0	0	16
	Total	1	26	0	1	0	0	0	28
Mandible/Face/Orthopantomogram	0–4	0	1	0	0	0	0	0	1
	5–8	0	0	0	0	0	0	0	0
	9–12	0	2	0	0	0	0	0	2
	13–16	0	1	0	0	0	0	0	1
	Total	0	4	0	0	0	0	0	4
Pelvis/Hip	0–4	0	1	0	0	0	0	0	1
	5–8	0	0	0	0	0	0	0	0
	9–12	1	2	0	0	0	0	0	3
	13–16	0	1	0	0	0	0	0	1
	Total	1	4	0	0	0	0	0	5
Shoulder girdle	0–4	3	2	0	0	0	0	0	5
	5–8	2	1	1	0	0	0	0	4
	9–12	4	5	0	0	0	0	0	9
	13–16	5	8	0	0	0	0	0	13
	Total	14	16	1	0	0	0	0	31
Spine (including soft tissue neck)	0–4	0	0	0	0	0	0	0	0
	5–8	0	0	0	0	0	0	0	0
	9–12	0	1	0	1	0	0	0	2
	13–16	0	2	0	0	0	0	0	2
	Total	0	3	0	1	0	0	0	4
Tibia/Fibula	0–4	2	6	0	1	0	1	0	10
	5–8	0	5	0	0	0	0	0	5
	9–12	0	2	0	0	0	0	0	2
	13–16	0	2	0	0	0	0	0	2
	Total	2	15	0	1	0	1	0	19
Wrist	0–4	0	0	0	0	0	0	0	0
	5–8	4	0	0	0	0	0	0	4
	9–12	12	14	1	2	2	1	0	32
	13–16	4	12	0	1	1	1	0	19
	Total	20	26	1	3	3	2	0	55
Total	0–4	21	47	3	4	1	3	1	80
	5–8	32	49	1	2	2	3	0	89
	9–12	46	98	1	8	2	6	0	161
	13–16	39	114	1	8	4	1	1	168
	Total	138	308	6	22	9	13	2	498

^a^ True positive.

^b^ True negative.

^c^ False positive.

^d^ False negative.

**TABLE 2:** Distribution of PIE categories.

PIE category	Frequency
True negative	308
True positive	138
False negative	22
Unsure	13
True positive/false negative	9
False positive	6
No participation	2

There were 26.5 (5.3%) FN scores, including 4.5 partial FNs. Table 3 presents the number of FN cases represented by each anatomical region. The five anatomical regions with the most FNs were the hand and fingers, wrist, elbow, ankle and forearm. FNs were recorded for all four age groups in the hand/fingers and elbow regions. FPs represented 6/498 (1.2%) of audited PIEs. The anatomical regions with FP comments were the hand/fingers, wrist, elbow, forearm and shoulder girdle. Patients aged 0–4 years had a higher proportion of FP cases (3/80, 3.7%), whereas the proportion of FP cases for patients aged 5–8 years, 9–12 years and 13–16 years was 1/89 (1.12%), 1/161 (0.62%) and 1/168 (0.60%), respectively. The TP/FN category was further explored in Table 4. One TP/FN score occurred due to incorrect categorisation of the type of fracture in the ankle. The remaining TP/FN scores (8/9) occurred in imaging of the radius and ulna (forearm, wrist and elbow examinations) due to not identifying all pathologies where multiple pathologies were present.

**TABLE 3:** Frequency of false negatives by anatomical region.

Anatomical region	Frequency	Percentage of false negatives
Hand/Fingers	8	30.2
Wrist	4.5	17
Elbow	4	15.1
Ankle	3.5	13.2
Forearm	2.5	9.4
Foot/Toes	1	3.8
Knee	1	3.8
Tibia/Fibula	1	3.8
Spine (including soft tissue neck)	1	3.8

**TABLE 4:** Reason for true positive/false negative category preliminary image evaluations.

Anatomical region	*N*	Reason for true positive/false negative
Forearm	3	Alerted # midshaft radius and ulna. Missed #/subluxation distal ulna.
		Alerted # radius. Missed # ulna.
		Alerted # distal radius. Missed # ulna.
Wrist	3	Alerted # radius. Missed # ulna.
		Alerted # distal radius. Missed # ulnar styloid.
		Alerted # distal radius. Missed # ulnar styloid.
Elbow	2	Alerted # radial head. Report stated # radial neck and proximal ulna.
		Alerted effusion. Missed # radial head.
Ankle	1	Alerted Salter–Harris Type IV #. Report stated Salter–Harris Type II
Chest/Abdomen	0	
Femur	0	
Foot/Toes	0	
Hand/Fingers	0	
Humerus	0	
Knee	0	
Mandible/Face/Orthopantomogram	0	
Pelvis/Hip	0	
Shoulder girdle	0	
Spine (including soft tissue neck)	0	
Tibia/Fibula	0	

*Note:* The word ‘fracture’ has been replaced with the convention ‘#’ within the table.

## 4 Discussion

This is the first study to focus solely on a paediatric population in the investigation of radiographer PIE accuracy. The present study demonstrated that radiographers could maintain a high diagnostic accuracy when interpreting radiographic examinations of paediatric patients presenting with trauma. Subsequently, a PIE service may provide a useful complement to aid emergency clinicians in interpreting paediatric radiographs when a definitive radiologist report is unavailable. This may contribute to patient safety in paediatric EDs by reducing the risk of diagnostic errors arising from radiographic misinterpretation [2].

Several prior studies have reported similar levels of PIE accuracy in clinical practice in interpreting adult examinations or adult and paediatric examinations [2, 13, 15, 16, 17, 18]. This study's accuracy (93%) was the highest out of the compared studies (88%–92%) [2, 13, 15, 16, 17, 18]. PIE accuracy did not appear to vary greatly between paediatric age groups, which was consistent with the results from a previous clinical study investigating the performance of emergency clinicians in interpreting paediatric skeletal radiographs [9]. The sensitivity in this study (84%) was within the range reported in prior studies (71%–94%) [2, 13, 15, 16, 17, 18]. The high specificity (98%) was also consistent with that of previous literature (91%–98%) [2, 13, 15, 16, 17, 18] and may be attributable to the low abnormality prevalence observed in clinical practice [20]. As the interpretation of paediatric radiographs is generally considered to be more complex [9, 10], one may suspect that a study focussed on paediatric examinations would demonstrate lower accuracy compared to studies that investigate adult populations. Some prior studies included paediatric examinations within their sample [2, 13, 15], which may contribute to the similarity in results compared to the present study. Interestingly, Petts et al. found that when compared to the overall study accuracy (92%), the paediatric population accuracy remained unchanged (92%), whilst the adult population accuracy was higher (95%) [2].

The literature has endeavoured to establish a benchmark accuracy for radiographers undertaking PIE to reduce adverse outcomes relating to discrepant radiographer X‐ray interpretation [13, 18]. Radiologists are considered the international ‘gold standard’ for the interpretation of radiographs [13, 21]. The present study demonstrated that radiographers do not share the same accuracy; however, comparison with the radiologist report is a markedly high benchmark given the considerable difference in radiographic interpretation experience and training that radiographers receive, compared with radiologists [13]. A minimum accuracy of 80% for radiographers undertaking PIE has been suggested, reflecting the typical performance expected of radiographers and junior doctors [22]. Other publications have recommended 90% accuracy as a benchmark for radiographers [22, 23, 24], with additional training undertaken if 90% accuracy is not achieved [23]. The results of this study exceed the 90% benchmark; this suggests that despite the challenges associated with interpreting paediatric radiographs, radiographers can maintain a high diagnostic accuracy.

As with previous studies, FN interpretations represented the majority of PIE discrepancies in the present study [13, 15]. The extremities, particularly the upper extremities, were regions that were frequently misinterpreted by radiographers in this study. Previous studies have also highlighted the extremities as a region that is frequently misinterpreted [9, 15, 25]. This may represent a possible area for targeted education, which has demonstrated the potential to improve radiographer PIE performance [26, 27]. Missed pathology in examinations with multiple injuries represented over one‐quarter of FN interpretations in the present study. This was similar to results reported in recent literature by Alexander‐Bates et al. [15] The term ‘Subsequent Search Miss’ has been used to describe errors relating to missed pathology in multiple injury examinations [28]. It is not uncommon for a radiograph to contain two or more abnormalities; hence, existing literature has highlighted the importance of ‘Satisfaction of Search’, that is, continuing to search for concomitant abnormalities once an initial abnormality is identified [28]. It was noted that all subsequent search miss errors in this study occurred in interpretation of the radius and ulna, which were captured under examinations of the forearm, wrist and elbow. Prior literature has concluded that continuing to search after identifying the first abnormality would enable further abnormalities to be identified in multi‐injury cases [15, 28]. These findings further highlight the importance of a systematic search strategy and ensuring satisfaction of search when interpreting radiographs [28].

### 4.1 Implications

This study contributes to the growing evidence base validating the utility of radiographer PIE as a patient safety mechanism to support emergency clinicians in interpreting radiographic examinations. Identification of significant pathology in radiographs and taking appropriate action to communicate these urgent findings and ensure patient safety are recognised as being within the radiographer's scope of practice and are mandated in the National Law for Australian radiographers [29]. Written communication of such findings, which can reduce miscommunication errors [30], is endorsed by the Medical Radiation Practice Board of Australia and the Australian Society of Medical Imaging and Radiation Therapy [29, 31]. Despite this, implementation of PIE systems has been slow [6, 26], potentially in part due to a lack of radiologist support and concerns about perceived role extension [3, 32, 33]. It was suggested that studies assessing the performance of PIE in clinical practice may assuage the concerns held by the Royal Australian and New Zealand College of Radiologists, whose official position remains that radiographer image interpretation is not the right solution [14, 32]. It may be useful to conduct a similar study investigating the paediatric population with a larger sample size and more rigorous data analysis, including an in‐depth analysis of FP and FN findings, which can further guide targeted training to improve the performance of radiographers.

### 4.2 Strengths and Limitations

There were several strengths and limitations to this study. The clinical study design and randomisation strategy were robust as they were based on a published audit methodology [15]. The study participants had varying levels of radiography experience; however, further demographic information was not collected. The participants of this study received X‐ray interpretation training through educational modules developed in‐house, which may limit generalisability to other departments. However, some studies investigating the existing radiographic interpretation skills of radiographers have demonstrated favourable levels of mean accuracy without formal education [16, 17, 34]. Whilst improved diagnostic accuracy is generally observed post‐training [16, 17, 26, 27], Lidgett et al. found no statistically significant difference in accuracy between trained and untrained radiographers in their pilot study [17]. Using a single radiologist report as the reference standard was another limitation due to the potential for errors in routine radiological reporting [13, 21]. In clinical practice, reporting of radiographic examinations was performed by different radiologists, who may have varying levels of experience. This introduced verification bias [35]. A double‐ or triple‐blinded consultant radiologist report for all examinations would be a more valid reference standard and could be generated given the retrospective study design; however, this may be unfeasible in a large audit. As data collection for the present study occurred secondary to the department's routine PIE audit, the number of paediatric studies selected each month was less than the recommended 100 examinations for a local clinical audit [15]. The recommended sample size requirement was met; however, auditing a larger sample size would enable more specific results when investigating relationships within age or anatomical region subgroups. Accuracy in interpreting specific anatomical regions was not compared due to the low number of examinations audited for certain regions. Analysis of PIE discrepancies by anatomical region resulted in a single pathology type (e.g., distal radius fracture) being grouped under different anatomical regions. This limited the accuracy with which trends in radiographer interpretation discrepancies could be depicted; therefore, future research should also analyse pathology types, so that radiographer education can be better targeted to areas requiring improvement [13].

## 5 Conclusion

This study undertook a 12‐month audit of PIE accuracy for paediatric trauma X‐rays. The present study demonstrated that radiographers could interpret paediatric trauma radiographs with a high level of diagnostic accuracy. Implementing a PIE service may aid emergency clinicians in interpreting paediatric radiographs in the absence of a timely radiologist report, thus enhancing patient safety in EDs that treat paediatric patients. The results identified potential for improvement in PIE accuracy in interpreting paediatric extremity X‐rays. Further research with a larger sample may enable a more in‐depth analysis of radiographer interpretation discrepancies by further defining areas for improvement to guide targeted X‐ray interpretation education.

## Ethics Statement

Ethics approval was granted by the Metro South Hospital and Health Service Human Research Ethics Committee (HREC/17/QPAH/796) and the Queensland University of Technology Research Governance and Integrity Team (7094). No personal identifying information was collected during this study.

## Conflicts of Interest

The authors declare no conflicts of interest.

## Data Availability

The data that support the findings of this study are available from the corresponding author upon reasonable request.
